# Removal of hexavalent chromium from wastewater by Cu/Fe bimetallic nanoparticles

**DOI:** 10.1038/s41598-021-90414-0

**Published:** 2021-05-25

**Authors:** Jien Ye, Yi Wang, Qiao Xu, Hanxin Wu, Jianhao Tong, Jiyan Shi

**Affiliations:** 1 Department of Environmental Engineering, College of Environmental and Resource Sciences, Zhejiang University, Hangzhou, 310058 China; 2 MOE Key Laboratory of Environment Remediation and Ecological Health, College of Environmental and Resource Science, Zhejiang University, Hangzhou, 310058 China

**Keywords:** Environmental sciences, Nanoscience and technology

## Abstract

Passivation of nanoscale zerovalent iron hinders its efficiency in water treatment, and loading another catalytic metal has been found to improve the efficiency significantly. In this study, Cu/Fe bimetallic nanoparticles were prepared by liquid-phase chemical reduction for removal of hexavalent chromium (Cr(VI)) from wastewater. Synthesized bimetallic nanoparticles were characterized by transmission electron microscopy, Brunauer–Emmet–Teller isotherm, and X-ray diffraction. The results showed that Cu loading can significantly enhance the removal efficiency of Cr(VI) by 29.3% to 84.0%, and the optimal Cu loading rate was 3% (wt%). The removal efficiency decreased with increasing initial pH and Cr(VI) concentration. The removal of Cr(VI) was better fitted by pseudo-second-order model than pseudo-first-order model. Thermodynamic analysis revealed that the Cr(VI) removal was spontaneous and endothermic, and the increase of reaction temperature facilitated the process. X-ray photoelectron spectroscopy (XPS) analysis indicated that Cr(VI) was completely reduced to Cr(III) and precipitated on the particle surface as hydroxylated Cr(OH)_3_ and Cr_x_Fe_1−x_(OH)_3_ coprecipitation. Our work could be beneficial for the application of iron-based nanomaterials in remediation of wastewater.

## Introduction

Chromium (Cr) is widely used in industry such as electroplating, leather tanning, and metallurgy^1,2^. During these industrial activities, Cr is inevitably released into groundwater and surface water^3^. Once entering into the environment, Cr exists in two stable states: hexavalent chromium (Cr(VI)) and trivalent chromium (Cr(III)). Trivalent chromium is non-toxic and present mainly in forms of Cr(OH)^2+^, Cr(OH)^2+^, and Cr(OH)_3_ in the environment. Since positively charged Cr(OH)^2+^ and Cr(OH)^2+^ can be absorbed onto the colloid and other media with negatively charged surface by electrostatic action, while Cr(OH)_3_ itself exists as precipitation, the mobility of Cr(III) in the environment is weak^4^. Compared with Cr(III), Cr(VI) (mainly exists as HCrO_4_^−^ and CrO_4_^2−^) is known to be carcinogenic, mutagenic, and teratogenic, and much more mobile in the environment^5,6^. In order to minimize the harm of Cr(VI), reduction of Cr(VI) to Cr(III) is one of the most common methods^7,8^.

Nanoscale zerovalent iron (nZVI) is a promising nanomaterial for removing pollutant from wastewater and has been used for Cr(VI) treatment in groundwater^9–12^. Removal of Cr(VI) involves (1) adsorption of Cr(VI) on the nanoparticles’ surface, (2) reduction of Cr(VI) to Cr(III), and (3) coprecipitation of Fe(III)-Cr(III) (oxy)hydroxides, as follows^13–15^:

1 $$ 3{\text{Fe}}^{0} + \, 2{\text{CrO}}_{4}^{2 - } + \, 16{\text{H}}^{ + } \to 3{\text{Fe}}^{2 + } + \, 2{\text{Cr}}^{3 + } + \, 8{\text{H}}_{2} {\text{O}} $$

2 $$ 3{\text{Fe}}^{2 + } + {\text{ CrO}}_{4}^{2 - } + \, 8{\text{H}}^{ + } \to \, 3{\text{Fe}}^{3 + } + {\text{ Cr}}^{3 + } + \, 4{\text{H}}_{2} {\text{O}} $$

3 $$ x{\text{Cr}}^{3 + } + \left( {1 - x} \right){\text{Fe}}^{3 + } + 2{\text{H}}_{2} {\text{O}} \to {\text{Cr}}_{x} {\text{Fe}}_{1 - x} {\text{OOH}} + 3{\text{H}}^{ + } $$

However, due to the high reactivity of nZVI, iron oxide film is easily formed on its surface which prevents the further contact with contaminants, especially in neutral and alkaline conditions^16,17^.

In recent years, loading another catalytic metal onto the surface of nZVI has been found to effectively alleviate the passivation of nZVI^16,18,19^. In bimetallic particles, the catalytic metal, such as Pd^20,21^, Ni^22,23^, and Cu^18,24^, can provide more reactivity sites and promote electron transfer on nanoparticles’ surface. Zhou et al.^25^ proved that the introduction of Ni to nZVI could prevent the aggregation of nZVI and improve the Cr(VI) removal efficiency. Hu et al.^26^ found that the loading of Cu could not only enhance the removal rate of Cr(VI) but also increase the thickness of the oxidation film and the oxidation state of iron, which meant higher removal capacity per unit weight of nZVI. Compared with other catalytic metals, Cu is an essential trace element for human health, and its cost is also lower. Therefore, Cu/Fe bimetallic nanoparticles show a strong application prospect in water treatment^27,28^. Recent studies have pointed out that (1) catalytic metals can enhance the oxidation of nZVI during the interaction with pollutants through the formation of galvanic cells; (2) catalytic metals can facilitate the generation of activated atomic hydrogens which lead to the enhancement of reduction of pollutants and corrosion of nZVI^29–31^. Therefore, to better understand the removal performance and mechanism of Cu/Fe bimetallic nanoparticles, the removal of Cr(VI) by Cu/Fe bimetallic nanoparticles was investigated in this study.

Cu/Fe bimetallic nanoparticles were prepared by chemical reduction in aqueous solutions and their reactivity towards Cr(VI) in wastewater was examined. The main aims of this study were to (1) investigate the Cr(VI) removal efficiency by bimetallic nanoparticles at different experiment conditions; (2) clarify the removal mechanism of Cr(VI) by Cu/Fe bimetallic nanoparticles. Results of this study could provide a theoretical basis for the application of iron-based nanomaterials in environmental remediation.

## Materials and methods

### Materials and chemicals

Ferric chloride hexahydrate (FeCl_3_∙6H_2_O), iron powder (100 mesh), anhydrous ethanol and cupric nitrate (Cu(NO_3_)_2_) were purchased from Sinopharm Chemical Reagent Co., Ltd, Shanghai, China. Potassium borohydride (KBH_4_) and polyvinylpyrrolidone (PVP, K29-32) were purchased from Aladdin Bio-Chem Technology Co., Ltd, Shanghai, China.

Hexavalent chromium wastewater was prepared by the extraction of soil sampled from a decommissioned chemical plant in Hangzhou, China (120° 18′ E, 30° 22′ N). After air-dried and sieved to less than 1 mm, the soil samples (50 g) were extracted by deionized water (1 L) for 18 h. The supernatant was then filtrated and stored as the Cr(VI) stock solution. Elemental concentrations of heavy metals were determined by an atomic absorption spectrometer (AAS, MKII M6, Thermo Electron, USA). The basic properties of the chromium wastewater (pH = 8.32 ± 0.10) were shown in Table 1.

**Table 1:** The concentrations of heavy metals in chromium wastewater (mg L^−1^).

Cr(VI)	Total Cr	Pb	Cu	Cd
1453.7 ± 6.3	1453.8 ± 8.3	3.24 ± 0.08	0.12 ± 0.01	0.04 ± 0.01

### Preparation of Cu/Fe bimetallic nanoparticles

The Cu/Fe bimetallic nanoparticles were prepared through a modified chemical reduction method reported before^26,32,33^. Briefly, 1.35 g FeCl_3_∙6H_2_O and 0.5 g PVP (K29-32) were dissolved in 100 mL ethanol–water solution (30% v/v) in a three-necked flask. After mechanical stirring under N_2_ environment for 10 min, 100 mL 0.2 M KBH_4_ (dissolved in 1% NaOH) was added dropwise by a constant pressure separation funnel. Ferric ions were reduced to zero-valent iron by the following reaction:

4 $$ {\text{4Fe}}^{{3 + }} {\text{ + 3BH}}_{{4}}^{ - } {\text{ + 9H}}_{{2}} {\text{O }} \to {\text{ 4Fe}}^{{0}} \downarrow {\text{ + 3H}}_{{2}} {\text{BO}}_{{3}}^{ - } {\text{ + 12H}}^{ + } {\text{ + 6H}}_{{2}} \uparrow $$

After being washed with deionized water and anhydrous ethanol for three times, the iron particles were dispersed in 100 mL oxygen-free water. Then, the desired doses of 1000 mg L^−1^ Cu(NO_3_)_2_ solution (2.8, 8.4, 14.0, 28.0 mL) were added dropwise for the preparation of bimetallic nanoparticles with the corresponding copper loading rates (1%, 3%, 5%, and 10%). After mechanical stirring under N_2_ environment for 30 min, the products were washed with anhydrous ethanol for three times. The nZVI and Cu/Fe bimetallic nanoparticles were sealed storage.

### Characterization of Cu/Fe bimetallic nanoparticles

The morphology of nZVI and Cu/Fe bimetallic nanoparticles was characterized by transmission electron microscope (JEM 1200EX, Hitachi, Japan) operating at 200 kV. Gas absorption operation was carried out for the determination of particles’ specific surface area in a surface area analyzer (Tristar II 3020, Micromeritics, USA). X-ray diffraction (XRD) was performed in a X-ray diffractometer (D/max-rA, Rigaku, Japan) with Cu Kα radiation (λ = 0.154 nm). The scan range was set from 10° to 90° at 40 kV and 40 kV. X-ray photoelectron spectroscopy was operated on a X-ray photoelectron spectrometer (Escalab 250Xi, Thermo Fisher Scientific, USA) with 250 W Mg Kα radiation.

### Batch experiments

Cr(VI) removal experiments were performed in 250 mL conical flasks with an oscillation frequency of 400 rpm. Each flask contained 0.01 g nZVI or Cu/Fe bimetallic nanoparticles and 100 mL Cr(VI) wastewater, which was diluted from the Cr(VI) stock solution (1453.7 mg L^−1^). After 1, 2, 5, 10, 20, 30, 60 min of reaction, 2 mL solution was sampled and filtered through a 0.22 μm membrane filter. The concentrations of Cr(VI) in the solutions were determined using the diphenylcarbohydrazide method. The absorbance of Cr(VI)-diphenylcarbohydrazide product was measured on a UV–vis spectrometer (UV-1800, Shimadzu, Japan). The removal efficiency (%) of Cr(VI) was calculated by the following Eq. ^34^:

5 $$ {\text{Removal}}\;{\text{efficiency}}\;\left( \% \right) = \left( {1{-}C_{i} /C_{0} } \right) \times 100 \, \% $$

where C_i_ is the residual Cr(VI) concentration and C_0_ is the initial Cr(VI) concentration in the filtered solutions.

## Results and discussion

### Characterization of Cu/Fe bimetallic nanoparticles

**Figure 1:**
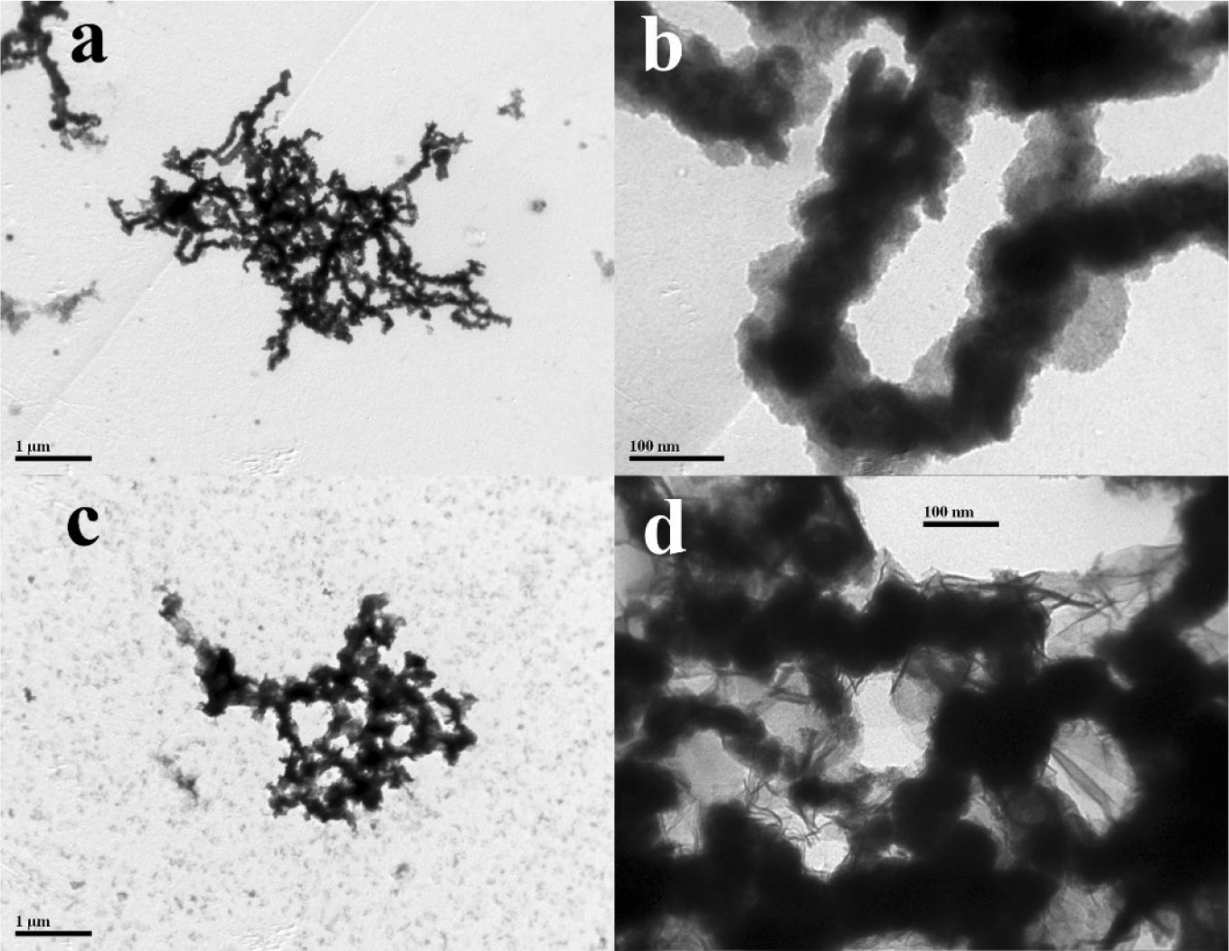
TEM images of nZVI (**a**,**b**) and Cu/Fe bimetallic nanoparticles (**c**,**d**).

**Figure 2:**
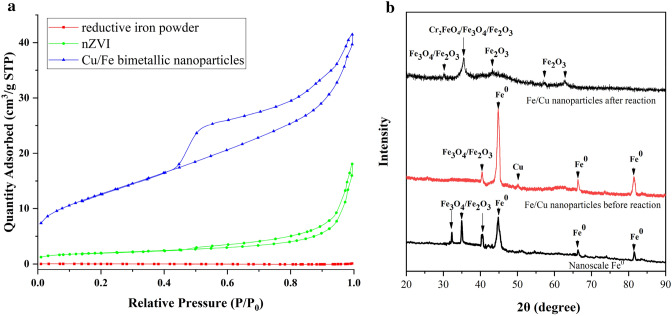
The adsorption–desorption isotherms of reductive iron powder, nZVI, and Cu/Fe bimetallic nanoparticles (**a**); XRD patterns of nZVI and Cu/Fe bimetallic nanoparticles (**b**).

The nZVI (Fig. 1a) and Cu/Fe bimetallic nanoparticles (Fig. 1c) were found to be mostly spherical in shape and both aggregated as a chain-like structure in the TEM images. The nZVI (Fig. 1b) and Cu/Fe particles (Fig. 1d) have similar particle sizes, both in the range of 50–100 nm. The strong agglomeration of nanoparticles can also be observed by SEM (Fig. S1). It can be found by SEM–EDS that copper is homogeneously distributed on the surface of iron particles (Fig. S2). The aggregation of particles can be mainly caused by the magnetism, electronic interactions between the metals and hydrogen bonds associated to surface groups^35^. The Brunauer-Emmet-Teller (BET) isotherm was used to determine the specific surface area of ordinary iron powder, nZVI and Cu/Fe bimetallic nanoparticles, respectively (Fig. 2a). The results showed that the specific surface area of nZVI (9.07 m^2^ g^−1^) was much higher than that of ordinary iron powder (0.15 m^2^ g^−1^). Copper loading can further increased the specific surface area of nZVI fivefold to 45.92 m^2^ g^−1^. This indicated that loading of Cu significantly reduced the agglomeration of nZVI. As observed from the XRD patterns of nZVI and Cu/Fe bimetallic nanoparticles (Fig. 2b), a diffraction peak occurred at 44.8° both in nZVI and Cu/Fe bimetallic nanoparticles, which corresponded to the (110) facet of bcc iron (JCPDS no. 06-0696)^25^. In addition, other diffraction peaks between 30° and 40° was also observed in nZVI, indicating the oxidation of iron and the formation of iron oxide (FeO) crystalline phases in nZVI (JCPDS no. 19-0629, 33–0664)^36^. However, such diffraction peaks missed in Cu/Fe bimetallic nanoparticles (Fig. 2b). Compared with nZVI, the stronger diffraction peak at 44.8° and lacking of peaks between 30° and 40° implied that loading of Cu on the surface of nZVI could effectively reduce the oxidation of iron. A diffraction peak at 50.4° was observed in the pattern of Cu/Fe bimetallic nanoparticles, which indicated that (200) facet of fcc copper (JCPDS no. 04–0836) was loaded on the surface of the particles^27^. In addition, the bimetallic nanoparticles after reaction with Cr(VI) was analyzed by XRD patterns (Fig. 2b). It was found that after the reaction, the diffraction peaks of Fe^0^ basically disappeared, and chromium and iron mainly existed in the form of oxides.

### Effect of Cu loading rate

**Figure 3:**
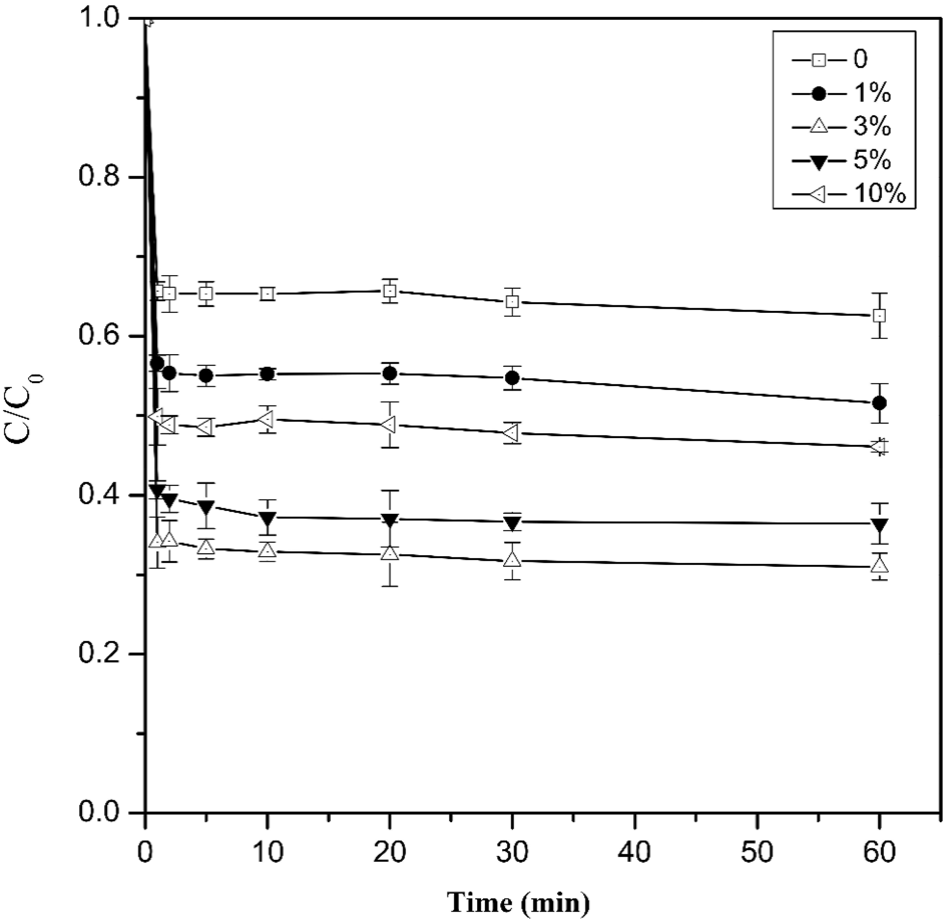
Effect of copper loading rates on Cr(VI) removal by Cu/Fe bimetallic nanoparticles. Error bars indicate the standard deviation of the mean (n = 3).

The effect of Cu loading rates (1%, 3%, 5%, 10%) on the removal efficiency of Cr(VI) (100 mg L^−1^) was investigated with batch experiments (Fig. 3). The Cr(VI) concentration decreased with increasing reaction time and the copper loading significantly increases the efficiency of Cr(VI) removal compared with nZVI. When Cu/Fe bimetallic nanoparticles with loading rates of 0, 1 and 3% were added into the 100 mg L^−1^ Cr(VI) solution for 60 min, their Cr(VI) removal efficiency could reach 37.47%, 48.46% and 68.94%, respectively. Bransfield et al.^32^ found that for Cu loading below 1 monolayer equivalent (~ 10 μmol Cu/g Fe), the deposition of Cu occurred onto the iron surface. When the Cu loading was greater than 1 monolayer, Cu deposition occurred predominantly onto metallic copper that had already been deposited before. Accordingly, the thickness of copper layer was assumed to increase linearly as the loading rates increased from 1 to 10% in this study. Due to the strong oxidizing ability of Cr(VI), the metallic copper on the iron surface could be oxidized by Cr(VI) if the copper layer is not thick enough (Eq. (6))^26^.

6 $$ 3{\text{Cu}} + 2{\text{CrO}}_{4}^{2 - } + 10{\text{H}}^{ + } \to 2{\text{Cr}}\left( {{\text{OH}}} \right)_{3} + 3{\text{Cu}}^{2 + } + 2{\text{H}}_{2} {\text{O}} $$

Therefore, an appropriate increase in Cu loading can enhance its catalytic ability. However, the removal efficiency of Cr(VI) declined as the Cu loading rate increased continuously to 5% and 10% (Fig. 3). This could be resulted from the aggregates of Cu nanoparticles and the shedding of Cu layer at the excessive Cu dosages^18,37^. Studies showed that excessive Cu was easy to be dropped off from the iron surface by outside force (e.g., agitation or fluidization), which resulted in the loss of catalytic ability^30^. In addition, excessive Cu loading resulted in a decrease of the Fe hydroxide amount on the surface of the particles. Due to the strong adsorption capacity of Fe hydroxide, the decrease of Fe hydroxide reduced the adsorption capacity of the nanoparticles to Cr(VI), which consequently declined the Cr(VI) removal efficiency.

### Effect of initial pH

**Figure 4:**
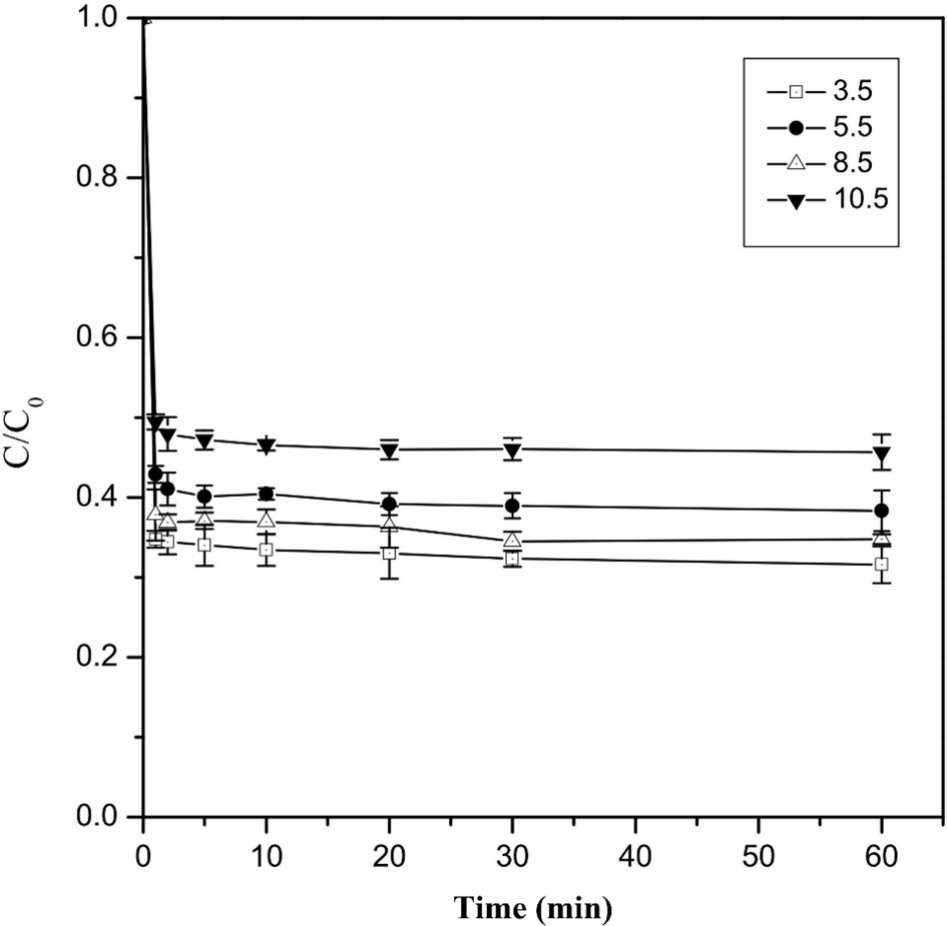
Effect of initial pH on Cr(VI) removal by Cu/Fe bimetallic nanoparticles. Error bars indicate the standard deviation of the mean (n = 3).

The removal efficiency of Cr(VI) by Cu/Fe bimetallic nanoparticles (3%) at initial pH of 3.5, 5.5, 8.5, 10.5 was investigated (Fig. 4). The removal efficiency of Cr(VI) was highest at pH of 3.5, reaching 69.40%, and slightly decreased to 61.65% and 63.23% when pH increased to 5.5 and 8.5, respectively. However, the removal efficiency of Cr(VI) decreased significantly from 69.40 to 55.34% as the pH increased from 3.5 to 10.5. This suggested that acidic conditions are more favorable for Cr(VI) removal by Cu/Fe nanoparticles. One possible reason is that under acidic conditions, the H^+^ in solution can dissolve the iron oxide film formed on the surface of the nanoparticles according to the Eq. (3), which increased the exposure of the particle surface active site, thus improving the removal efficiency of Cr(VI). While under alkaline conditions, the OH^-^ in solution can react with iron to produce an iron oxide passivation layer covering the surface of the nanoparticles, occupying the particle surface active sites and inhibiting the reduction reaction^38^. In addition, the competition between OH^-^ and Cr(VI) species (CrO_4_^2−^ and Cr_2_O_7_^2−^) at alkaline conditions also suppressed the removal efficiency. Similarly, Mortazavian et al.^39^ also found that the removal of aqueous Cr(VI) by activated carbon supported nZVI also showed a higher efficiency at pH of 4.0. The authors explained that the increasing H^+^ concentration allowed the reaction of the Eqs. (1) and (2) to proceed to the positive direction, so that more Cr(VI) was reduced to Cr(III).

### Effect of initial Cr(VI) concentration

**Figure 5:**
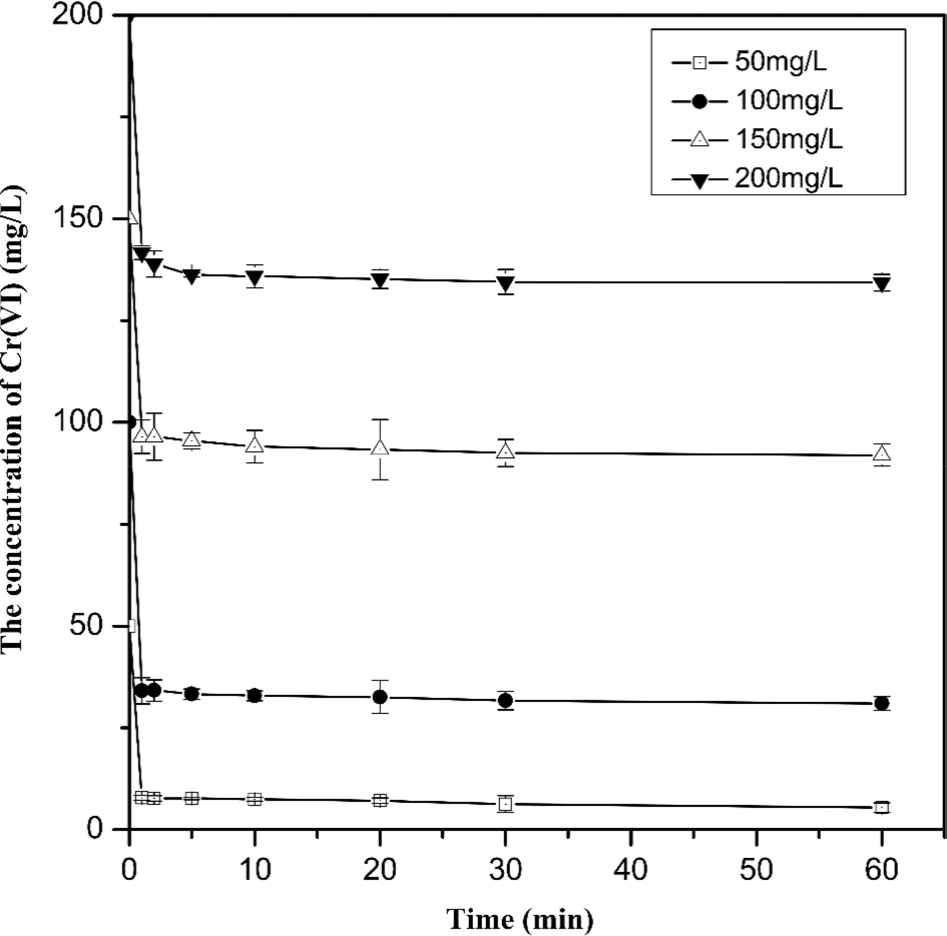
Effect of initial Cr(VI) concentrations on Cr(VI) removal by Cu/Fe bimetallic nanoparticles. Error bars indicate the standard deviation of the mean (n = 3).

In this study, the removal of Cr(VI) was investigated at initial Cr(VI) concentrations (pH = 8.32 ± 0.10) of 50, 100, 150 and 200 mg L^−1^, respectively, with Cu/Fe bimetallic nanoparticles (3%) applied at 0.1 g L^−1^ (Fig. 5). It can be found that the efficiency of Cr(VI) removal by Cu/Fe bimetallic nanoparticles decreased with the increase of the initial Cr(VI) concentration. When the initial Cr(VI) concentration was 50 mg L^−1^, the removal efficiency of Cr(VI) reached 89.2% after 60 min of reaction, and when the initial concentration increased to 100, 150, and 200 mg L^−1^, the corresponding removal efficiencies decreased to 68.9%, 40.7%, and 32.8%, respectively. According to the Eqs. (1) and (2), 1 mol of Fe^0^ can theoretically provide 3 mol of electrons for reducing Cr(VI)^40^.Therefore, ideally 0.1 g L^−1^ of Cu/Fe bimetallic nanoparticles in an anaerobic environment can fully reduce about 93 mg L^−1^ Cr(VI). However, in this study, the removal amounts of Cr(VI) was 68.92, 61.10 and 65.68 mg L^−1^ at initial Cr(VI) concentrations of 100, 150 and 200 mg L^−1^, respectively. This may be due to the fact that some of the Fe^0^ was oxidized so that the amount of electrons provided by Fe for Cr(VI) reduction decreased. In addition, we found that the amount of Cr(VI) removal decreased with the initial Cr(VI) concentration (Fig. 5). A possible reason is that with the increase of the initial Cr(VI) concentration, Cr(VI) rapidly occupied the active site on the nZVI surface and formed a Fe(III)-Cr(III) (oxy)hydroxides passivation layer, which inhibited the release of Fe^2+^ and the reduction of Cr(VI) subsequently^41,42^.

### Kinetics of Cr(VI) removal

**Figure 6:**
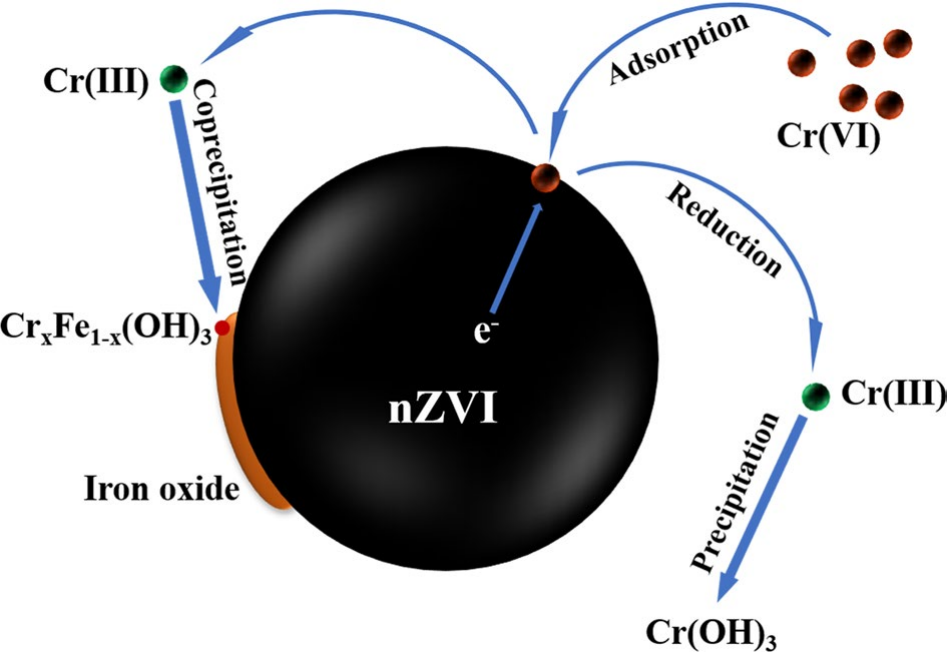
Proposed mechanism for the removal of Cr(VI) using nZVI.

The Cr(VI) concentration in the wastewater decreased drastically in the first 2 min (Fig. 3). The reduction and removal of Cr(VI) by nZVI was carried out in two stages: (1) the Cr(VI) was firstly adsorbed on the particle surface of nZVI and (2) reduced then by internal electron transfer (Fig. 6). The pseudo-first-order kinetic model and pseudo-second-order kinetic model were used to analyze the kinetics of removal of Cr(VI)^43,44^:

7 $$ {\text{ln}}\left( {\frac{{\text{C}}}{{{\text{C}}_{{0}} }}} \right){\text{ = - k}}_{{{\text{obs}}}} {\text{t}} $$

8 $$ \frac{{\text{t}}}{{{\text{Q}}_{{\text{t}}} }}{ = }\frac{{1}}{{{\text{k}}_{{2}} {\text{Q}}_{{\text{e}}}^{{2}} }}{ + }\frac{{\text{t}}}{{{\text{Q}}_{{\text{e}}} }} $$

where C_0_ and C (mg L^−1^) is the concentration of Cr(VI)initially and at different time, k_obs_ (min^−1^) is the observed first-order rate coefficiency, Q_t_ (mg g^−1^)is adsorption capacity at time ‘t’, k_2_ (g mg^−1^ min^−1^) is the pseudo-second-order rate constant, and Q_e_ (mg g^−1^) is equilibrium adsorption capacity.

The k_obs_ can be obtained by fitting ln(C/C_0_) to the reaction time (Figs. S3-S5, Table S1). When the Cu loading rate increased from 0 to 1% and 3%, the k_obs_ increased from 0.0008 min^−1^ to 0.0009 min^−1^ and 0.0016 min^−1^, respectively. As the loading rate increased continuously to 5% and 10%, the k_obs_, however, decreased to 0.0015 min^−1^ and 0.0009 min^−1^ (Fig. S3). This also agreed with the observation that the excessive Cu loading was not conducive to the reaction of nZVI with Cr(VI). The k_obs_ values obtained in this study were small compared with other studies^45,46^. This may be because that the Cr(VI)-containing wastewater used in the present study was extracted from chromium-contaminated soil rather than formulated with potassium dichromate. Cr(VI)-containing wastewater contained other ions such as lead, cadmium and copper, which could affect the removal efficiency. The effects of several common ions (Pb^2+^, Cu^2+^,Cd^2+^, Ca^2+^, and Mg^2+^) in Cr(VI)-polluted wastewater were investigated (Fig. S9). These ions were found to reduce the Cr(VI) removal efficiency of Cu/Fe bimetallic nanoparticles. On the one hand, the presence of these ions could compete for the reaction sites on the surface of the iron particles^47^. On the other hand, certain ions (such as Ca^2+^) could result in nZVI aggregation and reduce the removal efficiency^27^. Moreover, the solution pH was alkaline (pH = 8.32 ± 0.10), which inhibited the reaction of Cu/Fe nanoparticles with Cr(VI) under high pH conditions^48^. Compared with pseudo-first-order kinetic model, the regression values were higher and the calculated Q_e_ values are well matched with the experimental values (Fig S6, Table S2). This indicated that the removal process of Cr(VI) by Cu/Fe bimetallic nanoparticles followed chemisorption involving the electrons exchange between nanoparticles and Cr(VI)^43^.

Similarly, the k_obs_ can be obtained by analyzing the relationship between ln(C/C_0_) and reaction time under different initial pH conditions (Fig. S4, Table S1). It can be found that the k_obs_ decreased with increasing pH. The k_obs_ was 0.0016 min^−1^ when pH was 3.5, and the k_obs_ decreased to 0.0014 min^−1^ when pH increased to 5.5 and 8.5. When pH was 10.5, the k_obs_ decreased by 62% to 0.0006 min^−1^ compared to pH 3.5. The different reaction rates at the corresponding pH could be explained by the Cr(VI) speciation and the surface charges of the nanoparticles. At pH > 6.5, Cr(VI) speciation is predominantly CrO_4_^2−^, and at pH < 6.5, HCrO_4_^-^ is the dominant form. As the pH decreases, the reduction of hexavalent chromium becomes easier^25,41,49^. In addition, the equilibrium adsorption capacity Q_e_ also decreased with increasing pH (Fig. S7, Table S2). This might be related to electrostatic adsorption. The pH of zero point of charge (pH_zpc_) for Cu/Fe bimetallic nanoparticles was measured to be 4.6 (see Fig. S11 in the Supplemental Information), which means that the surface of the Cu/Fe bimetallic nanoparticles is positively charged when the pH is less than 4.6. Therefore, at the initial pH of 3.5, there was an electrostatic attraction between the Cu/Fe bimetallic nanoparticles and HCrO_4_^-^, which allowed the Cr(VI) to be more easily adsorbed on the surface of iron particles and promoted the reduction rate^39,50,51^.

The analysis of the Cr(VI) removal process at different initial Cr(VI) concentrations by a pseudo-first-order kinetic model revealed that the k_obs_ decreased as the initial concentration of Cr(VI) increased (Fig. S5, Table S1). When the concentration of Cr(VI) increased from 50 to 100, 150 and 200 mg L^−1^, the corresponding k_obs_ decreased from 0.0064 to 0.0016, 0.0008 and 0.0006 min^−1^, respectively. The k_obs_ at low Cr(VI) concentration (50 mg L^−1^) was almost 10 times higher than that at higher concentrations (200 mg L^−1^). Similar results were reported by Geng et al.^52^. When the initial concentration of Cr(VI) was low, the ratio of nanoparticles/Cr(VI) was relatively high, resulting in a relatively high probability of Cr(VI) contact with Cu/Fe bimetallic nanoparticles, which in turn accelerated the reaction rate. Different from the reaction rate, Q_e_ values of Cu/Fe bimetallic nanoparticles for Cr(VI) did not increase with the increase of the initial concentration of Cr(VI) (Fig S8, Table S2). The Q_e_ reached its maximum when the initial Cr(VI) concentration is 100 mg L^−1^, which was 689.6 mg g^−1^.

### Adsorption isotherms and thermodynamic study of Cr(VI) removal

Two common adsorption isotherm models (Langmuir and Freundlich) were adopted to study the removal of Cr(VI) by Cu/Fe bimetallic nanoparticles (3%) at different initial concentrations. The equations of Langmuir and Freundlich model are as follows^50^:

9 $$ \frac{{{\text{C}}_{{\text{e}}} }}{{{\text{q}}_{{\text{e}}} }}{ = }\frac{{{\text{C}}_{{\text{e}}} }}{{{\text{q}}_{{\text{m}}} }}{ + }\frac{{1}}{{{\text{K}}_{{\text{L}}} {\text{q}}_{{\text{m}}} }} $$

10 $$ {\text{lnq}}_{{\text{e}}} { = }\frac{{1}}{{\text{n}}}{\text{lnC}}_{{\text{e}}} {\text{ + lnK}}_{{\text{F}}} $$

where C_e_ is the concentration at equilibrium (mg L^−1^), q_e_ is the equilibrium adsorption capacity (mg g^−1^), q_m_ is the monolayer adsorption capacity (mg g^−1^), K_L_ is the Langmuir constant (L mg^−1^), K_F_ is the Freundlich constant (L g^−1^), and n is the Freundlich exponent.

Compared to the Freundlich model, the Langmuir model is a better description of the process of Cr(VI) removal by Cu/Fe bimetallic nanoparticles (Table 2). The squared correlation coefficient (R^2^) of the Langmuir model was closer to 1, which was much higher than the R^2^ of the Freundlich model. This phenomenon indicates that the process of Cr(VI) removal by Cu/Fe bimetallic nanoparticles is a monolayer adsorption, with Cr(VI) homogeneously distributed on the adsorption sites on the material surface^50,53^. The separation factor R_L_ is an important parameter of the Langmuir model, defining by the following equation:

11 $$ {\text{R}}_{{\text{L}}} { = }\frac{{1}}{{{\text{1 + K}}_{{\text{L}}} {\text{C}}_{{0}} }} $$

where K_L_ is the Langmuir constant (L mg^−1^), and C_0_ is the initial concentration of Cr(VI) solution. When R_L_ = 0, the process of isothermal adsorption is irreversible. The adsorption process is favorable when R_L_ is between 0 and 1, and the smaller the R value, the more favorable the process. When R_L_ = 1, the process is unfavorable. In our experiment, the values of R_L_ with different initial Cr(VI) concentrations were close to zero and decreased with increasing initial concentrations. This indicated that the adsorption process was favorable, and there was a strong affinity between Cr(VI) ions and Cu/Fe bimetallic nanoparticles^43,53^.

Thermodynamic studies describe the spontaneity of the reaction, the degree of system randomness, and the endothermic/exothermic condition of the reaction. The equations for calculating thermodynamics are as follows^43^:

12 $$ {\Delta G}^{{0}} {\text{ = - RTlnK}}_{{\text{c}}} $$

where ΔG^0^ is Gibbs free energy, R is universal gas constant (8.314 J mol^−1^ K^−1^), T is absolute temperature (K), and Kc is the distribution coefficient calculated by

13 $$ {\text{lnK}}_{{\text{c}}} { = - }\frac{{{\Delta H}^{{0}} }}{{{\text{RT}}}}{ + }\frac{{{\Delta S}^{{0}} }}{{\text{R}}} $$

14 $$ {\text{K}}_{{\text{C}}} { = }\frac{{{\text{q}}_{{\text{e}}} }}{{{\text{C}}_{{\text{e}}} }} $$

where ΔH^0^ is enthalpy change, ΔS^0^ is entropy change, q_e_ is the equilibrium adsorption capacity (mg g^−1^), and C_e_ is the Cr(VI) concentration at equilibrium (mg L^−1^).

The thermodynamic parameters for the removal of Cr(VI) were shown in Table S1. Negative ΔG values indicated that this process was spontaneous, and the values tended to decrease with the increase of temperature, suggesting that an appropriate increase in reaction temperature facilitated the process. Positive values of ΔH and ΔS revealed that this process was endothermic and the randomness degree of system increased. Similar results of adsorption/reduction thermodynamic process of Cr(VI) by nZVI have been reported by other researchers^54,55^.

**Table 2:** Parameters of isotherm models (298 K).

Isotherm model	C_0_	Parameters			
Langmuir		K_L_/L mg^−1^	q_m_/mg g^−1^	R^2^	R_L_
		0.526	644.33	0.990	
	50				0.0366
	100				0.0186
	150				0.0125
	200				0.0094
Freundlich		K_F_/L g^−1^	1/n	R^2^	
		403.83	0.102	0.572	

### XPS analysis

**Figure 7:**
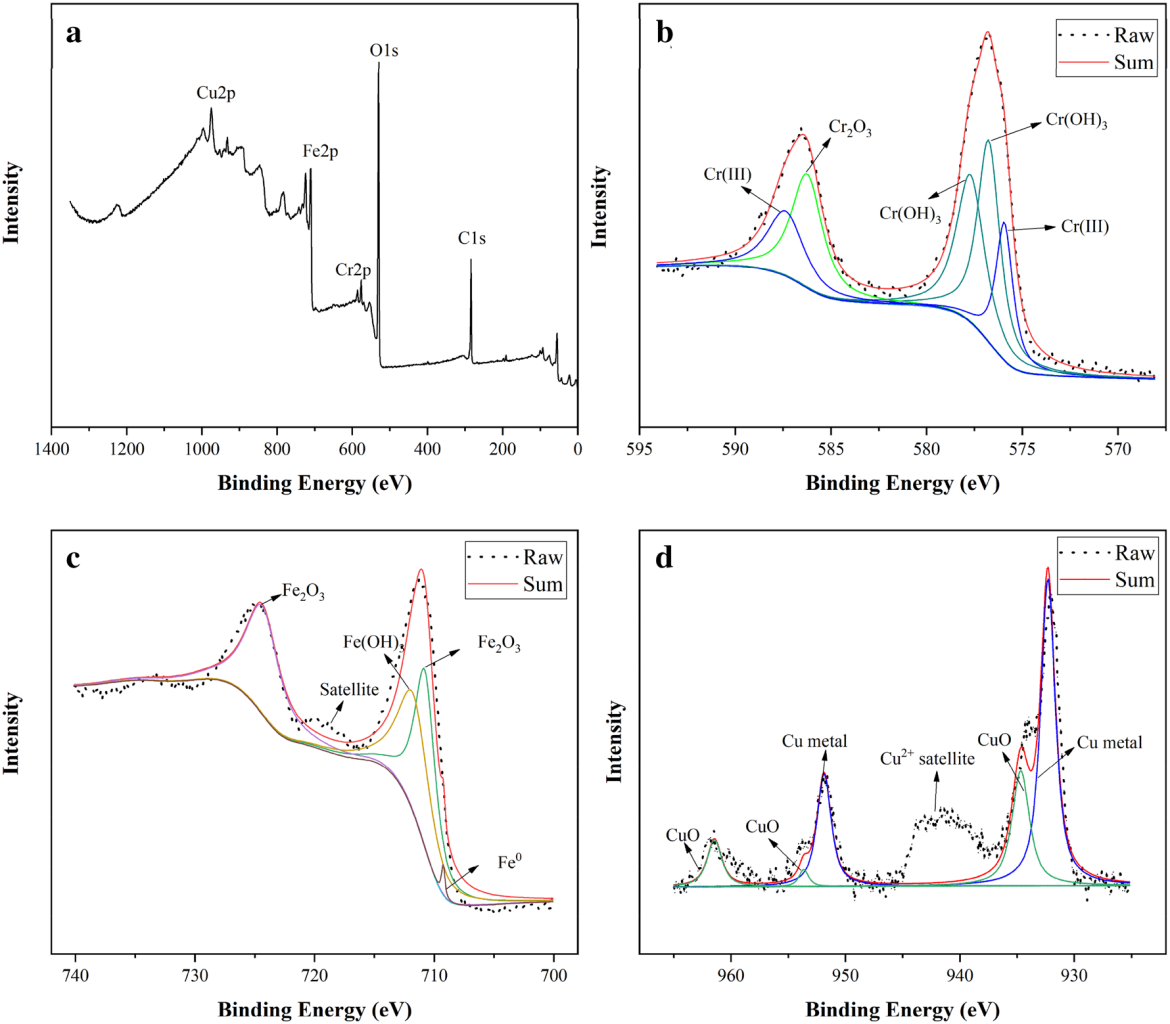
XPS spectra of Cu/Fe bimetallic nanoparticles after removal reaction with Cr(VI): (**a**) wide scan, (**b**) Cr 2p spectrum, (**c**) Fe 2p spectrum, (**d**) Cu 2p spectrum.

The products in the reduction of Cr(VI) by the Cu/Fe bimetallic nanoparticles were analyzed by XPS (Fig. 7a). The reaction products mainly consisted of Fe, Cr, Cu, O and C elements. The photoelectron peak for Cr at about 577 eV indicated that Cr could become a solid-phase deposit attached to the surface of Cu/Fe nanoparticles after the reaction^56^. The main way for Cr(VI) removal is the redox reaction of nZVI with Cr(VI), which produce Fe(III) and Cr(III). This process consumes H^+^, thus Fe(III) and Cr(III) can combine with OH^-^ to form Fe(OH)_3_, Cr(OH)_3_, or Cr_x_Fe_1-x_(OH)_3_ coprecipitation^57^. Since the Cu/Fe nanoparticle is a bimetal system, when the insoluble film forms on the surface of the iron particle, the electrons of nZVI and Cu can still transfers through the Cu layer to Cr(VI)^26^. Therefore, the reaction can continue as long as the Cu layer is not completely covered by the oxide film.

To further analyze the chemical compositions of Cr ,Fe and Cu, detailed XPS spectra for Cr 2p,Fe 2p and Cu 2p were carried out (Fig. 7b-d). The Cr 2p_3/2_ and Cr 2p_1/2_ photoelectron peaks at 577.0 eV and 586.8 eV represent the binding energies of Cr(OH)_3_, Cr(OH)O, and Cr_2_O_3_^48,58,59^. The spin–orbit splitting energy is 9.8 eV and Full-Width at Half-Maximum (FWHM) is 3.0 eV, indicating that Cr(III) is the dominant species of Cr on the particle surface^60,61^. The photoelectron peak for Cr(VI) was not detected in the reaction products. This proved that Cr(VI) was reduced to Cr(III) by interaction with Cu/Fe bimetallic nanoparticles and was eventually deposited on the particle surface. The Fe 2p_3/2_ and Fe 2p_1/2_ photoelectron peaks at 711.3 eV and 725.2 eV indicate the Fe(III) species in the product, with possible chemical structures of hydrated ferric oxide (FeOOH), magnetite (Fe_3_O_4_) or hematite (Fe_2_O_3_)^48^. Besides, there was a satellite peak between two dominant peaks, which indicated a shake-up process of Fe(II)^25^. In the study reported by Li et al.^62^, a photoelectron peak at 706.9 eV was observed after nZVI was exposed to Cr(VI) solution, which meant the existence of Fe^0^ on the surface of reaction products. In our study, few peak of Fe^0^ was detected in the Fe 2p spectra, indicating that the Fe^0^ in the Cu/Fe nanoparticles had been oxidized to Fe(III) after the reaction. Compared with the case studied by Li et al.^62^, it can be demonstrated that the reaction of nZVI with Cr(VI) generates oxide film on the surface, which affects the efficiency of Cr(VI) removal, while the loading of Cu can reduce the passivation of nZVI and make the reaction of nZVI with Cr(VI) more complete. The spectrum results of Cu 2p showed that Cu mainly existed in the form of Cu metal and CuO (Fig. 7d). This indicated that during the reduction of Cr(VI) by Cu/Fe bimetallic nanoparticles, Cu might also participate in the reaction, and part of the Cu^0^ was oxidized by Cr(VI). The standard electrode potential of Cr(VI) is 1.35 V (HCrO_4_^−^/Cr^3+^), Fe^0^ is -0.45 V (Fe^2+^/Fe^0^), and Cu is 0.34 V (Cu^2+^/Cu^0^). The potential difference of the reaction between Cr(VI) and Cu is 1.01 V. Thermodynamically, Cr(VI) is able to oxidize copper metal. Therefore, in the reaction process of Cr(VI) and Cu/Fe bimetallic nanoparticles, Cu not only acted as an electron transporter to transfer electrons from Fe^0^ to Cr(VI), but also acted as an electron donor to provide electrons for the reduction of Cr(VI).

## Conclusion

In this study, it was found that the loading of Cu can significantly increase the specific surface area of nZVI, while the degree of oxidation of Fe^0^ can be attenuated. The Cu/Fe bimetallic nanoparticles can effectively remove Cr(VI) from the solution, with the best removal capability at the Cu loading rate of 3%. The Cr(VI) removal capacity of nZVI increased from 374.7 to 689.4 mg g^−1^ with a copper loading rate of 3%. The initial pH and Cr(VI) concentration of the wastewater also affected the removal of Cr(VI) by Cu/Fe bimetallic nanoparticles, and the removal efficiency decreases with increasing pH and Cr(VI) concentration. Under acidic conditions (pH = 3.5), the equilibrium adsorption capacity of Cu/Fe bimetallic nanoparticles is 26% higher than that under alkaline conditions (pH = 10.5). After the reaction, the Cr(VI) in solution was reduced to Cr(III) and precipitated on the surface of the particles, while Fe^0^ was precipitated as an oxide of Fe(III). The process of Cr(VI) removal was found to be spontaneous, endothermic and favorable. This process followed pseudo-second-order kinetics, and chemisorption-couple reduction was the predominant mechanism of Cr(VI) removal. The results of this study could provide a theoretical basis for the application of iron-based nanomaterials in remediation of wastewater.

## Supplementary Information

Supplementary Information.
