# Supplementary material for: Removal of hexavalent chromium from wastewater by Cu/Fe bimetallic nanoparticles
Source: Sci Rep. 2021 May 25;11:10848. doi: 10.1038/s41598-021-90414-0 (PMC8149398; doi:10.1038/s41598-021-90414-0)
Supplement: Supplementary file 1 — Supplementary Information. [file 41598_2021_90414_MOESM1_ESM.docx]

**Supplemental Information**

**Removal of hexavalent chromium from wastewater by** **Cu/Fe bimetallic nanoparticles**

**Jien Ye ^1,2^, Yi Wang^1,2^, Qiao Xu^1,2^, Hanxin Wu^1,2^, Jianhao Tong^1,2^, Jiyan Shi ^1,2^***

1 Department of Environmental Engineering, College of Environmental and Resource Sciences, Zhejiang University, Hangzhou 310058, China;

2 MOE Key Laboratory of Environment Remediation and Ecological Health, College of Environmental & Resource Science, Zhejiang University, Hangzhou 310058, China;

*Corresponding author

Jiyan Shi

Tel.: +86-571-8898-2019; Fax: +86-571-8898-2019; E-mail: [shijiyan@zju.edu.cn](mailto:shijiyan@zju.edu.cn)

**Number of pages: 15**

**Number of tables: 3**

**Number of figures: 11**

**Contents:**

**Table S1** Kinetic parameters of the pseudo-first-order kinetic model.

**Table S2** Kinetic parameters of the pseudo-second-order kinetic model.

**Table S3** Thermodynamic parameters.

**Fig. S1** SEM images of nZVI (a) and Cu/Fe bimetallic nanoparticles (b).

**Fig. S2** SEM-EDX images of Cu/Fe bimetallic nanoparticles (10% Cu loading rate).

**Fig. S3** Effect of copper loading rates on pseudo-first-order kinetics of Cr(VI) reduction by Cu/Fe bimetallic nanoparticles.

**Fig. S4** Effect of initial pH on pseudo-first-order kinetics of Cr(VI) reduction by Cu/Fe bimetallic nanoparticles.

**Fig. S5** Effect of initial Cr(VI) concentrations on pseudo-first-order kinetics of Cr(VI) reduction by Cu/Fe bimetallic nanoparticles.

**Fig. S6** Effect of Cu loading rates on pseudo-second-order kinetics of Cr(VI) reduction by Cu/Fe bimetallic nanoparticles.

**Fig. S7** Effect of initial pH on pseudo-second-order kinetics of Cr(VI) reduction by Cu/Fe bimetallic nanoparticles.

**Fig. S8** Effect of initial Cr(VI) concentrations on pseudo-second-order kinetics of Cr(VI) reduction by Cu/Fe bimetallic nanoparticles.

**Fig. S9** Effect of co-existing ions on the removal of Cr(VI) by Cu/Fe bimetallic nanoparticles (initial Cr(VI) concentration: 100 mg L^-1^, pH = 3.5, Cu/Fe bimetallic nanoparticles: 0.1 g L^-1^, the asterisk (*) indicate the statistically significant difference among the different groups (p < 0.05)).

**Fig. S10** Effect of temperature on Cr(VI) removal by Cu/Fe bimetallic nanoparticles.

**Fig. S11** Zeta potential of nZVI and Cu/Fe bimetallic nanoparticles with different pH.

**Table S1** Kinetic parameters of the pseudo-first-order kinetic model

| Cu loading rate (wt%) | k_obs_ (min^-1^) | R^2^ |
| --- | --- | --- |
| 0 | 0.0008 | 0.8673 |
| 1% | 0.0009 | 0.8675 |
| 3% | 0.0016 | 0.8882 |
| 5% | 0.0015 | 0.5904 |
| 10% | 0.0009 | 0.8518 |
| Initial pH |  |  |
| 3.5 | 0.0016 | 0.9003 |
| 5.5 | 0.0014 | 0.7459 |
| 8.5 | 0.0014 | 0.6523 |
| 10.5 | 0.0006 | 0.5770 |
| Cr(VI) concentration (mg L^-1^) |  |  |
| 50 | 0.0064 | 0.9766 |
| 100 | 0.0016 | 0.8882 |
| 150 | 0.0008 | 0.7780 |
| 200 | 0.0006 | 0.4874 |

**Table S2** Kinetic parameters of the pseudo-second-order kinetic model

| Cu loading rate (wt%) | k_2_ (10^-3^g mg ^-1^min^-1^) | Q_e_ (mg g^-1^) | R^2^ |
| --- | --- | --- | --- |
| 0 | 4.12 | 374.5 | 0.998 |
| 1% | 3.06 | 483.1 | 0.998 |
| 3% | 6.83 | 689.7 | 0.999 |
| 5% | 10.70 | 636.9 | 0.999 |
| 10% | 4.15 | 540.5 | 0.995 |
| Initial pH |  |  |  |
| 3.5 | 6.99 | 684.9 | 0.999 |
| 5.5 | 8.02 | 617.3 | 0.999 |
| 8.5 | 7.80 | 653.6 | 0.998 |
| 10.5 | 13.21 | 543.5 | 0.999 |
| Cr(VI) concentration (mg L^-1^) |  |  |  |
| 50 | 6.46 | 446.4 | 0.999 |
| 100 | 6.78 | 689.6 | 0.999 |
| 150 | 5.83 | 581.4 | 0.999 |
| 200 | 7.83 | 657.9 | 0.999 |

**Table S3** Thermodynamic parameters

|  | Temperature (K) | ΔG  (kJ mol^-1^) | ΔH  (kJ mol^-1^) | ΔS  (kJ mol^-1^ K^-1^) |
| --- | --- | --- | --- | --- |
| Cu loading rate: 3%  Initial Cr(VI): 100mg L^-1^ | 298 | -7.69 | 19.42 | 0.0908 |
|  | 303 | -8.01 |  |  |
|  | 308 | -8.60 |  |  |


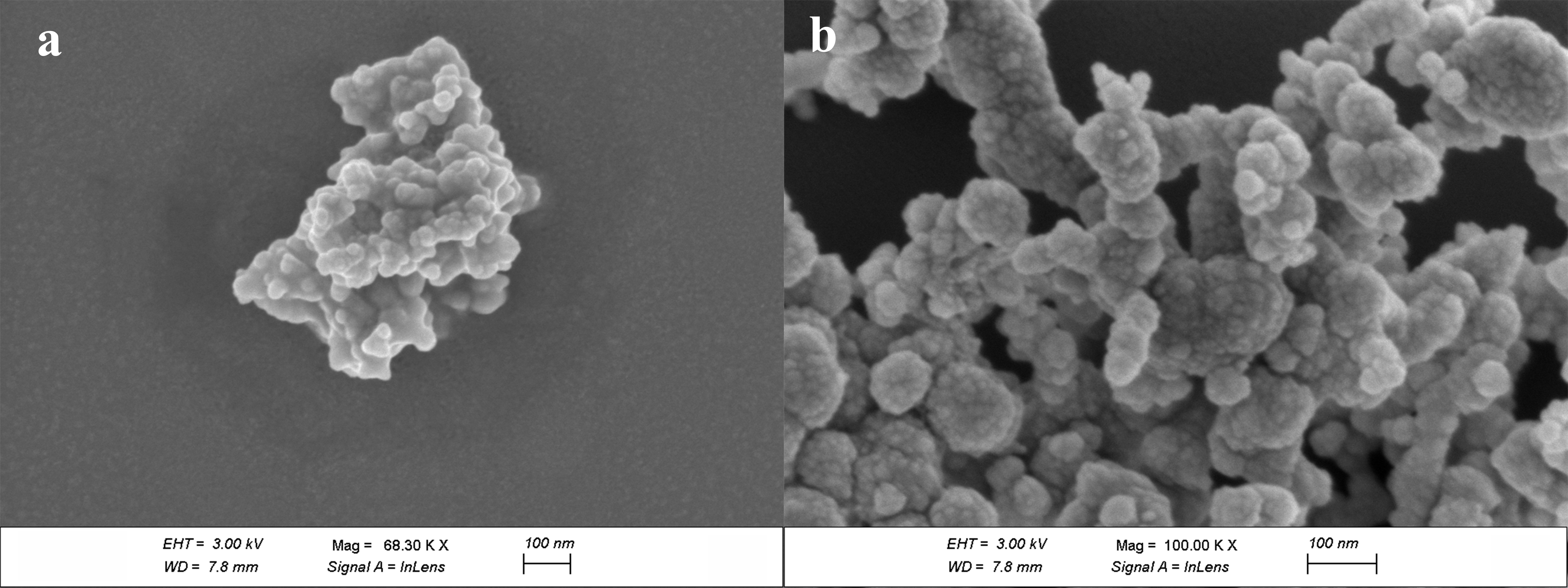


Fig. S1 SEM images of nZVI (a) and Cu/Fe bimetallic nanoparticles (b)


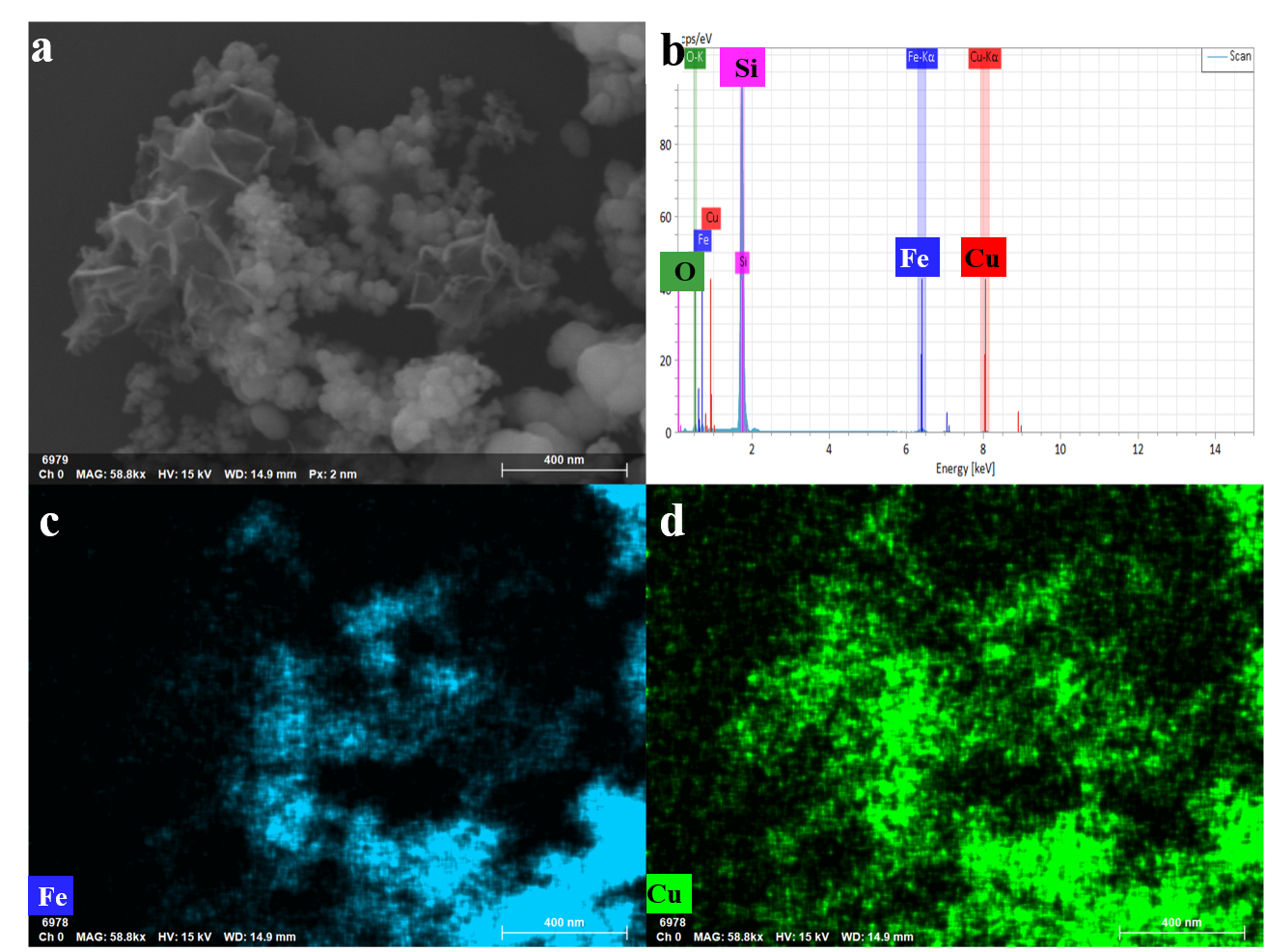


Fig. S2 SEM-EDX images of Cu/Fe bimetallic nanoparticles (10% Cu loading rate)


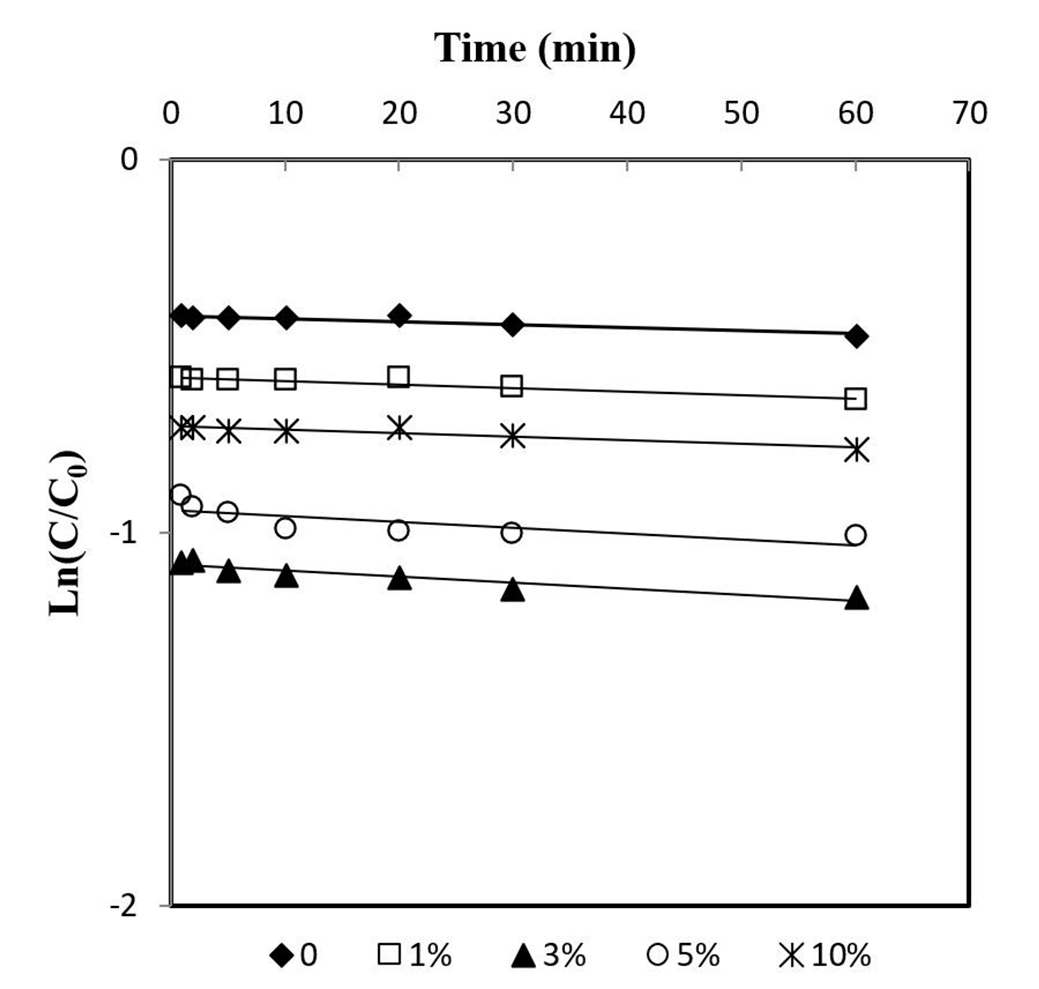


Fig. S3 Effect of copper loading rates on pseudo-first-order kinetics of Cr(VI) reduction by Cu/Fe bimetallic nanoparticles


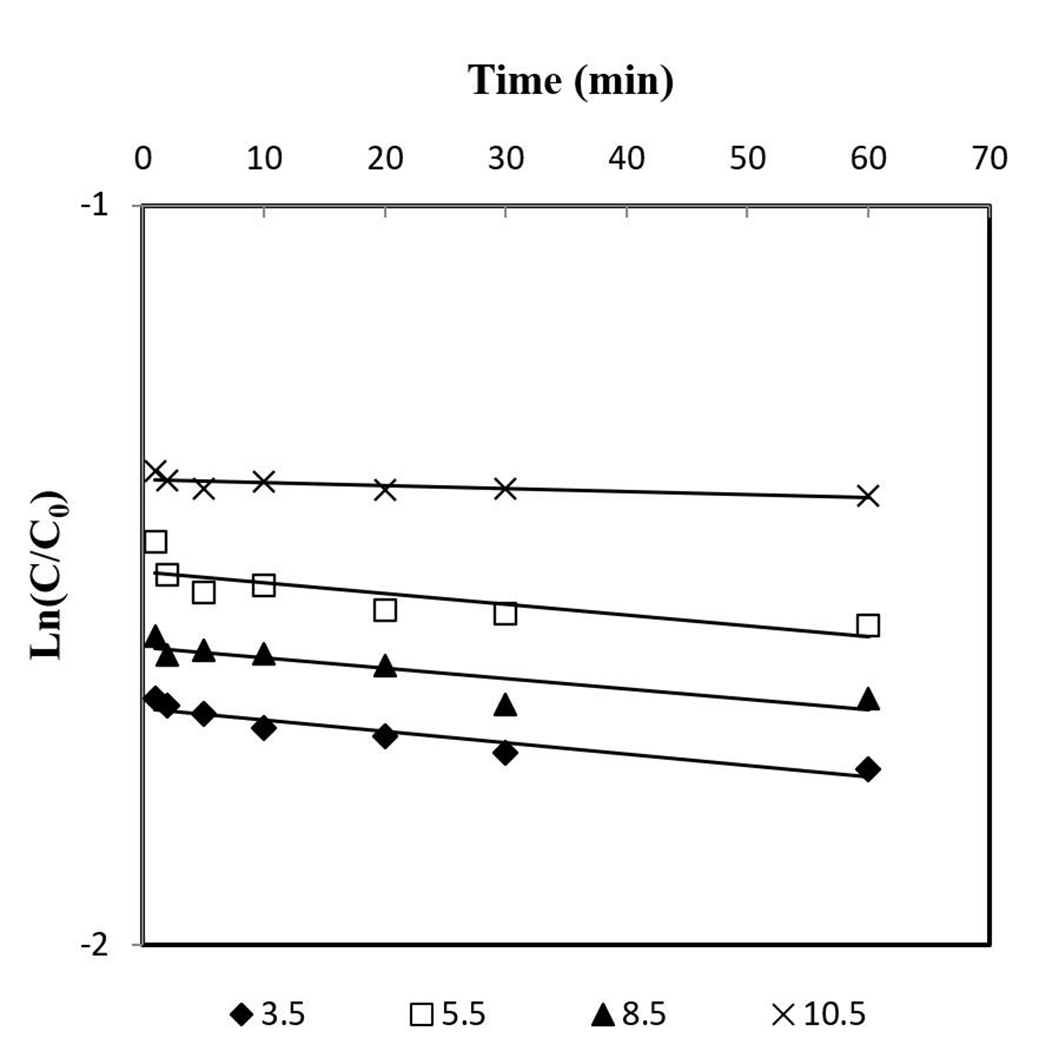


Fig. S4 Effect of initial pH on pseudo-first-order kinetics of Cr(VI) reduction by Cu/Fe bimetallic nanoparticles


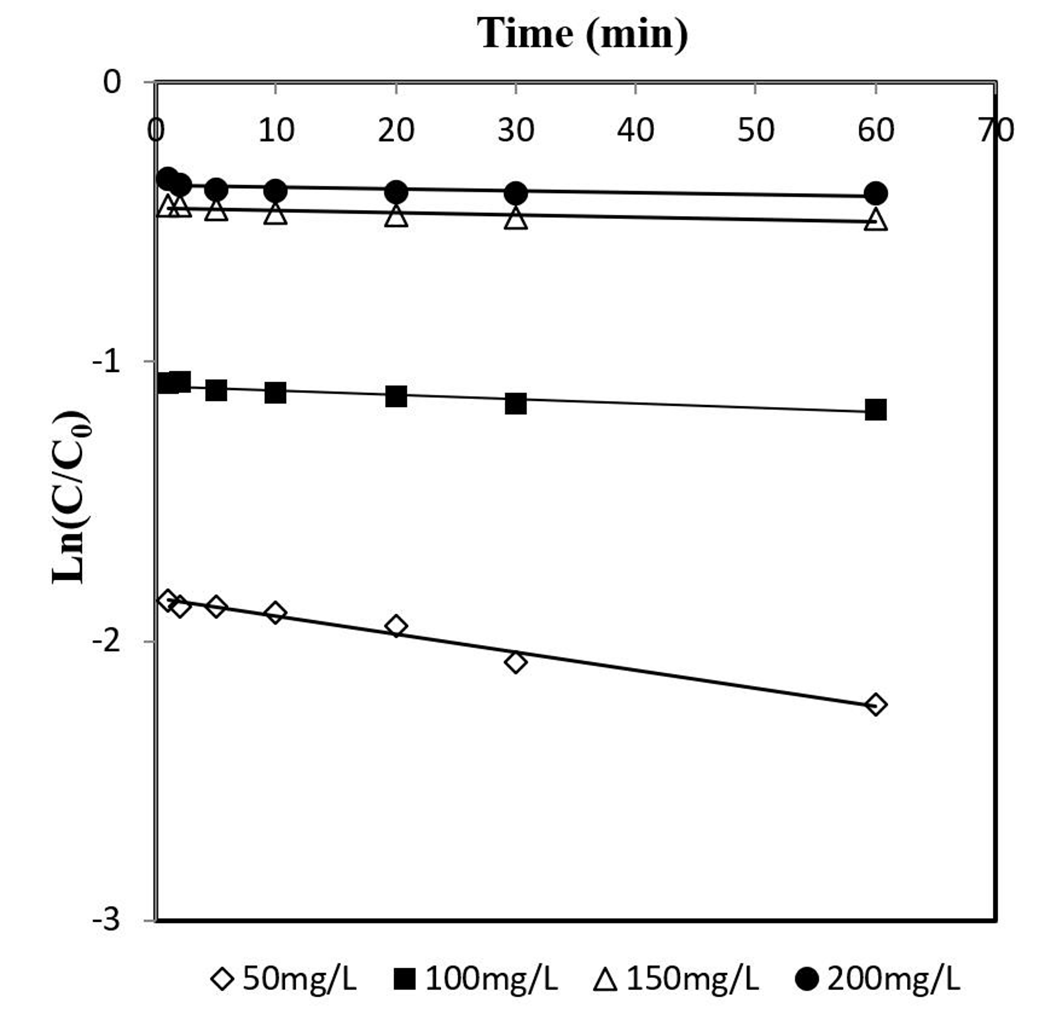


Fig. S5 Effect of initial Cr(VI) concentrations on pseudo-first-order kinetics of Cr(VI) reduction by Cu/Fe bimetallic nanoparticles





Fig. S6 Effect of Cu loading rates on pseudo-second-order kinetics of Cr(VI) reduction by Cu/Fe bimetallic nanoparticles



 Fig. S7 Effect of initial pH on pseudo-second-order kinetics of Cr(VI) reduction by Cu/Fe bimetallic nanoparticles



 Fig. S8 Effect of initial Cr(VI) concentrations on pseudo-second-order kinetics of Cr(VI) reduction by Cu/Fe bimetallic nanoparticles





Fig. S9 Effect of co-existing ions on the removal of Cr(VI) by Cu/Fe bimetallic nanoparticles (initial Cr(VI) concentration: 100 mg L^-1^, pH = 3.5, Cu/Fe bimetallic nanoparticles: 0.1 g L^-1^, the asterisk (*) indicate the statistically significant difference among the different groups (p < 0.05)).





Fig. S10 Effect of temperature on Cr(VI) removal by Cu/Fe bimetallic nanoparticles





Fig. S11 Zeta potential of nZVI and Cu/Fe bimetallic nanoparticles with different pH
